# Supplementary material for: Carbon dioxide electroreduction on single-atom nickel decorated carbon membranes with industry compatible current densities
Source: Nat Commun. 2020 Jan 30;11:593. doi: 10.1038/s41467-020-14402-0 (PMC6992760; doi:10.1038/s41467-020-14402-0)
Supplement: Supplementary file 1 — Supplementary Information [file 41467_2020_14402_MOESM1_ESM.pdf]

Supplementary Information

Carbon dioxide electroreduction on single-atom nickel decorated carbon membranes with industry compatible current densities

Yang et al.

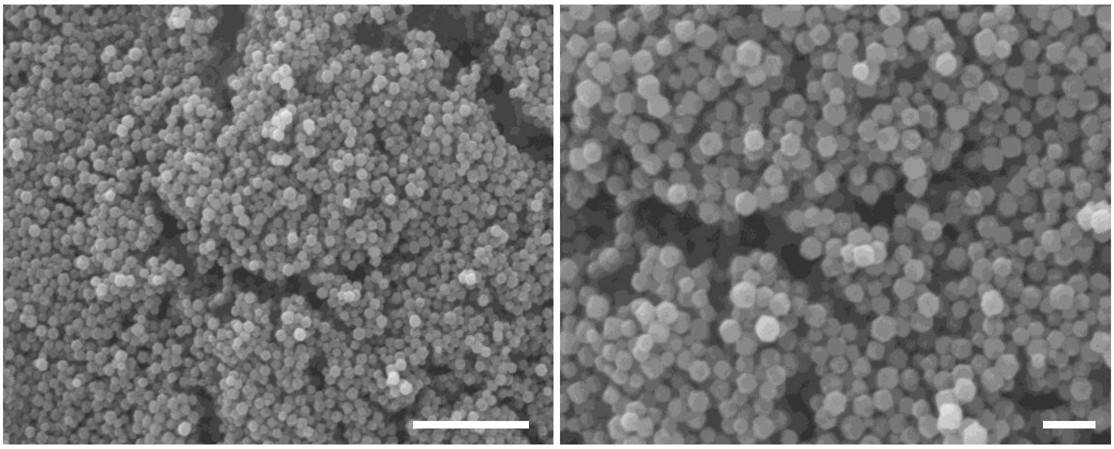

Supplementary Figure 1. SEM characterizations. SEM images of pure ZIF-8. Scale bars, 1 μm (left), and 200 nm (right).

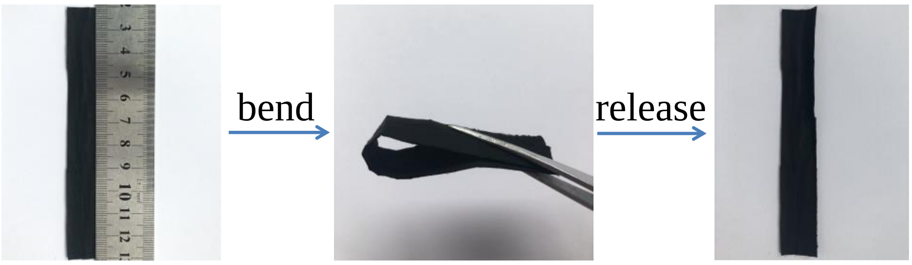

Supplementary Figure 2. **Mechanical tests.** Digital images of a piece of flexible NiSA/PCFM sample for 200 times bending tests.

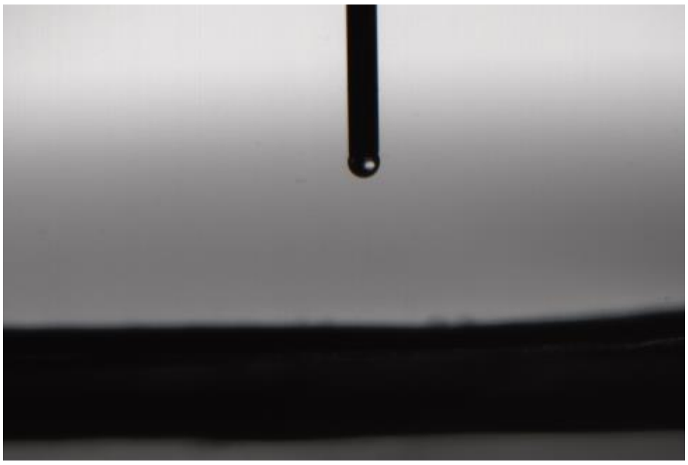

Supplementary Figure 3. Water contact tests. The water contact angles of NiSA/PCFM before spraying Nafion solution. The water contact angle of NiSA/PCFM catalyst was nearly 0°, showing the hydrophilic property.

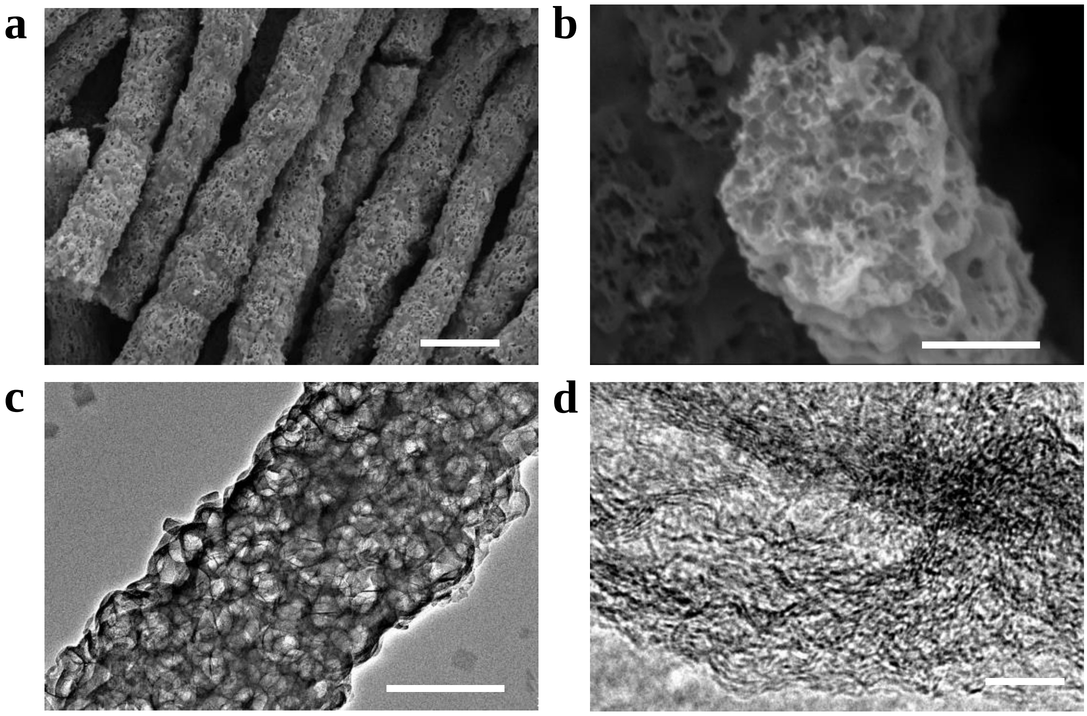

Supplementary Figure 4. Structure characterizations of NiSA/PCFM. SEM (a–b) and TEM (c–d) images of NiSA/PCFM, respectively. Scale bars, 2 μm (a), 0.5 μm (b), 0.5 μm (c) and 5 nm (d).

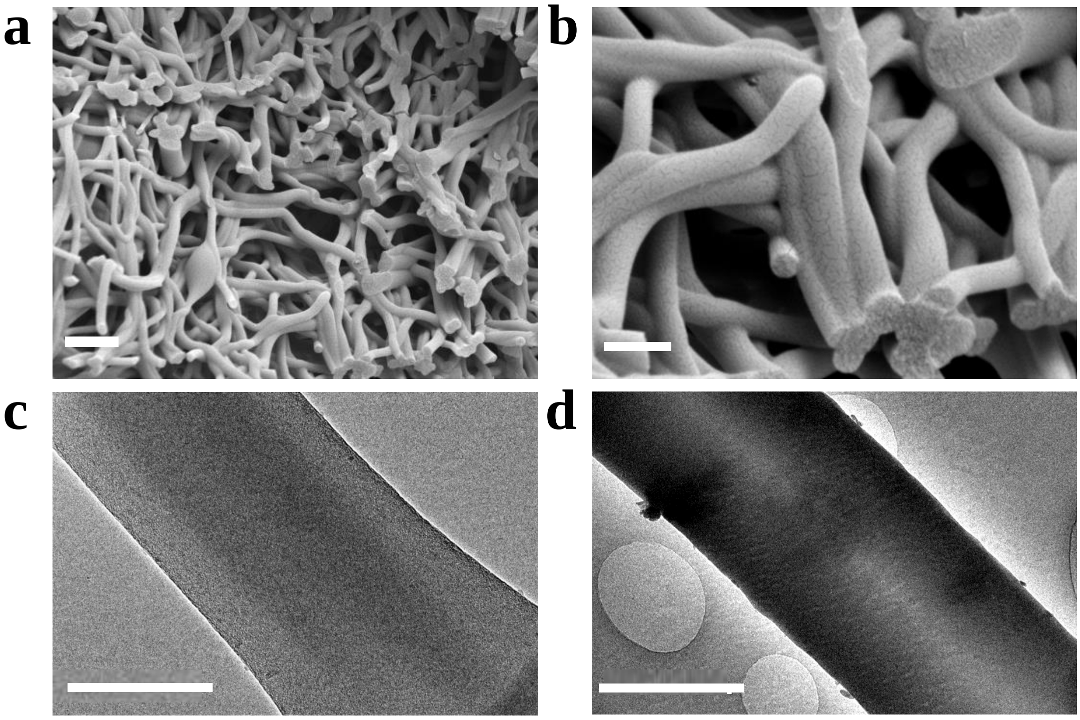

Supplementary Figure 5. Structure characterizations of NiSA/CFM. SEM (a–b) and TEM (c–d) images of NiSA/CFM, respectively. Scale bars, 1 μm (a), 500 nm (b), 500 nm (c) and 500 nm (d).

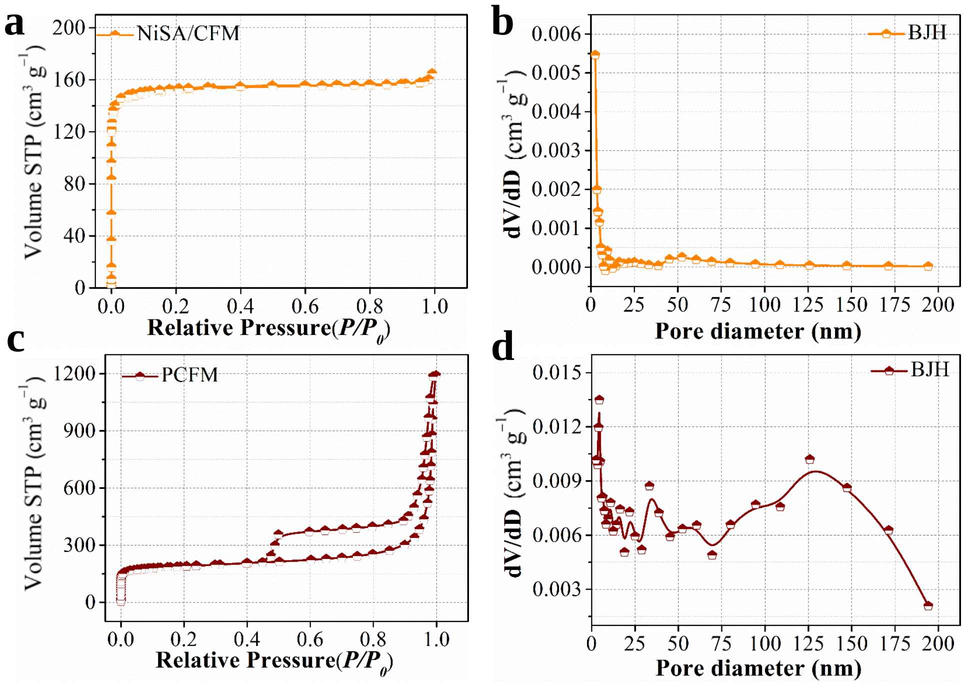

Supplementary Figure 6. Characterizations of Porous structure. N_2 sorption isotherms and pore size distributions of NiSA/CFM (a–b) and PCFM (c–d), respectively.

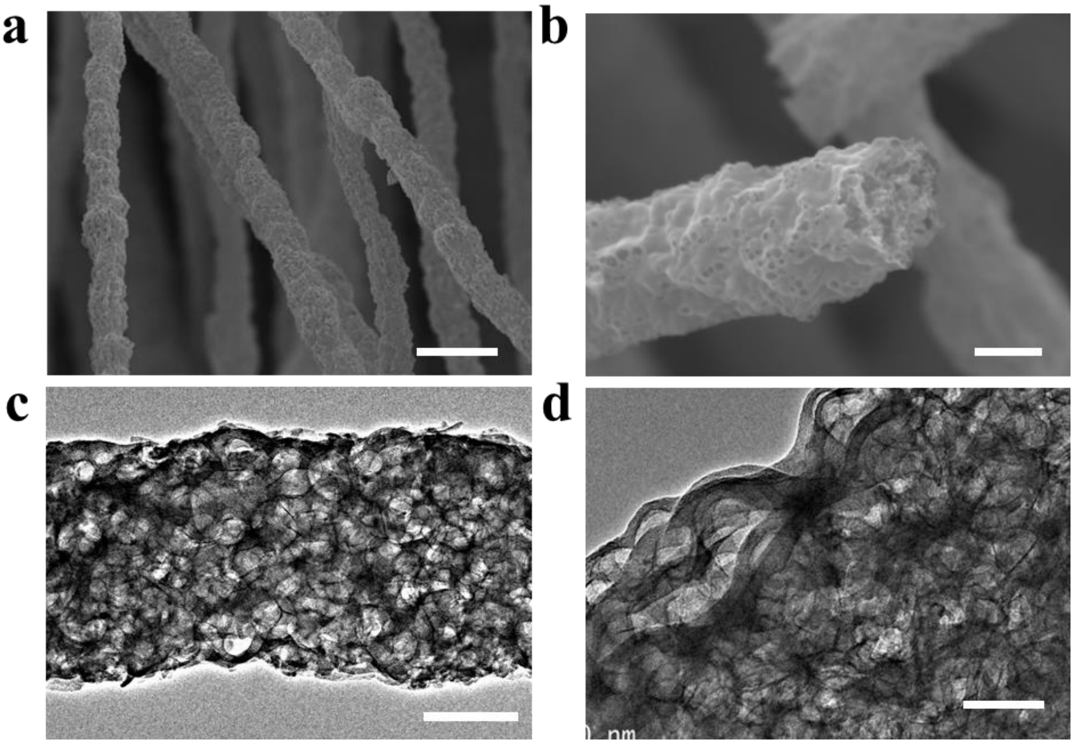

Supplementary Figure 7. **Structure characterizations of PCFM.** SEM (a–b) and TEM (c–d) images of PCFM, respectively. Scale bars, 2 μm (a), 500 nm (b), 200 nm (c) and 100 nm (d).

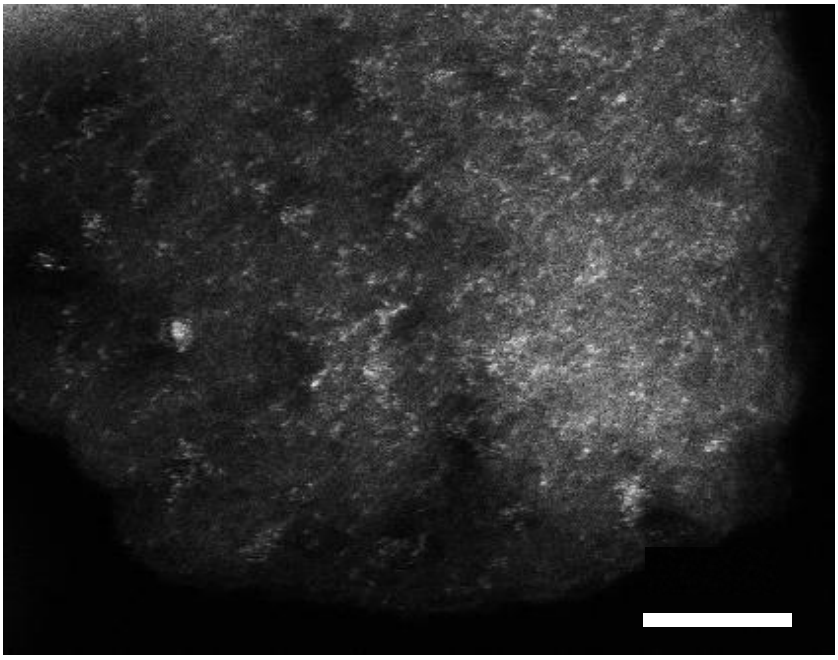

Supplementary Figure 8. **Atom-scale characterization.** Aberration-corrected HAADF-STEM image of NiSA/CFM, those white dots are supposed to be Ni single atoms. Scale bar 2 nm.

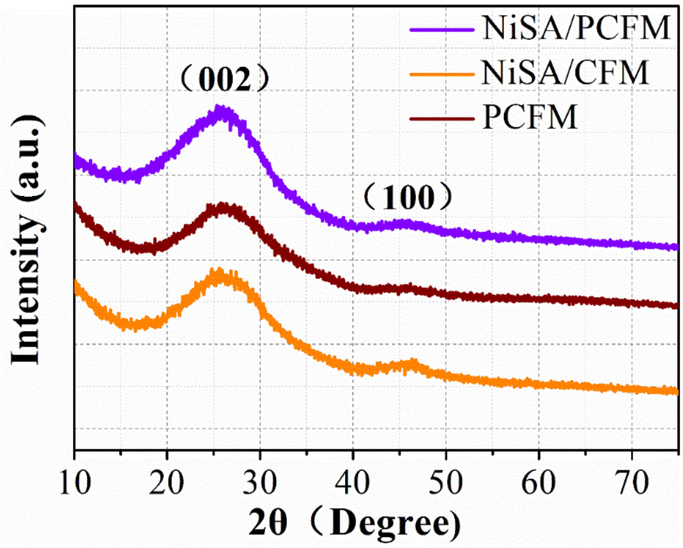

Supplementary Figure 9. **XRD characterization.** XRD spectra of three samples.

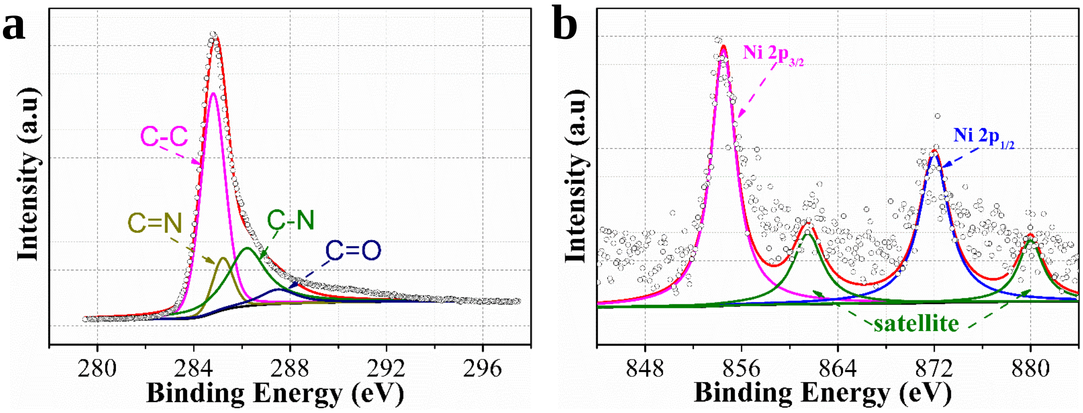

Supplementary Figure 10. **XPS spectra of NiSA/PCFM.** C *1s* (a) and Ni *2p* (b) XPS spectra.

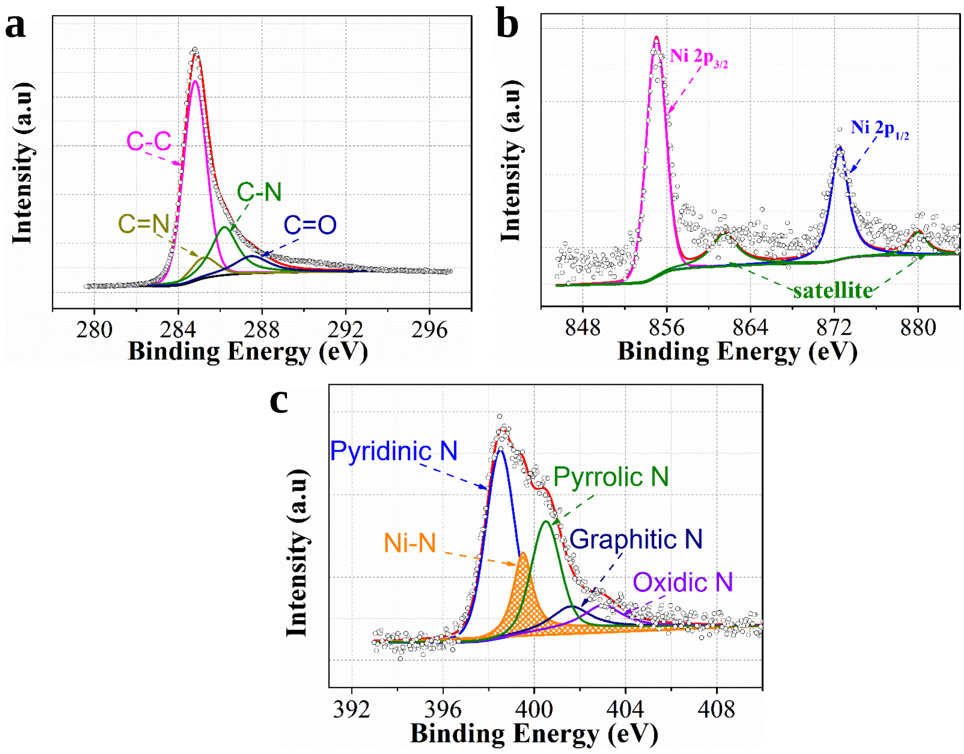

Supplementary Figure 11. **XPS spectra of NiSA/CFM.** C *1s* (a), Ni *2p* (b) and N *1s* (c) XPS spectra.

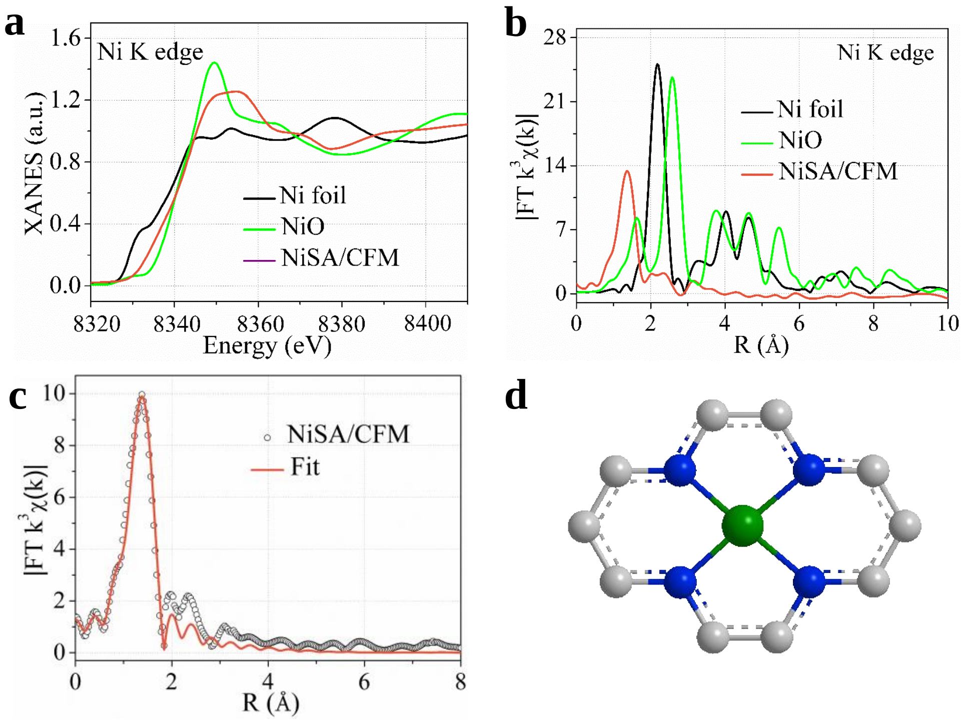

Supplementary Figure 12. XAFS spectra of NiSA/CFM. XANES (a) and EXAFS (b) spectra at the Ni K-edge of Ni foil, NiO and NiSA/CFM; Fitting for EXAFS data of NiSA/CFM (c–d).

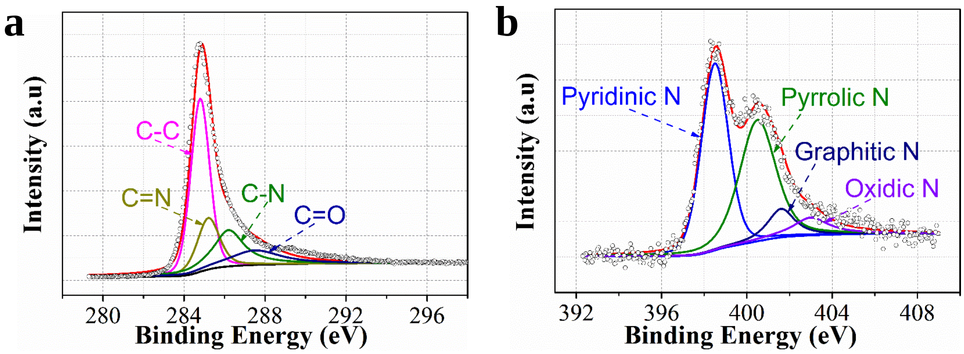

Supplementary Figure 13. **XPS spectra of PCFM.** C *1s* (a) and N *1s* (b) XPS spectra.

a

b

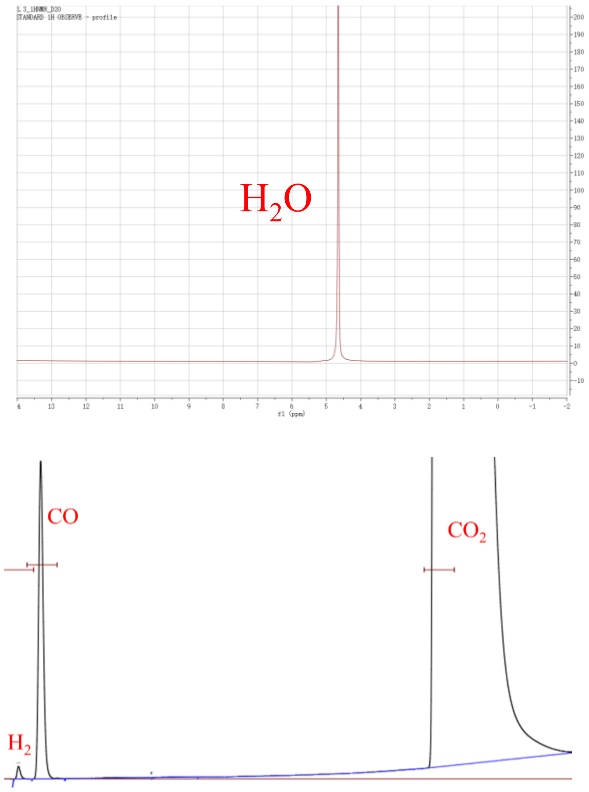

Supplementary Figure 14. **Qualitative detection of reduction products.** ^{1}H NMR spectra and gas chromatogram of products from NiSA/PCFM.

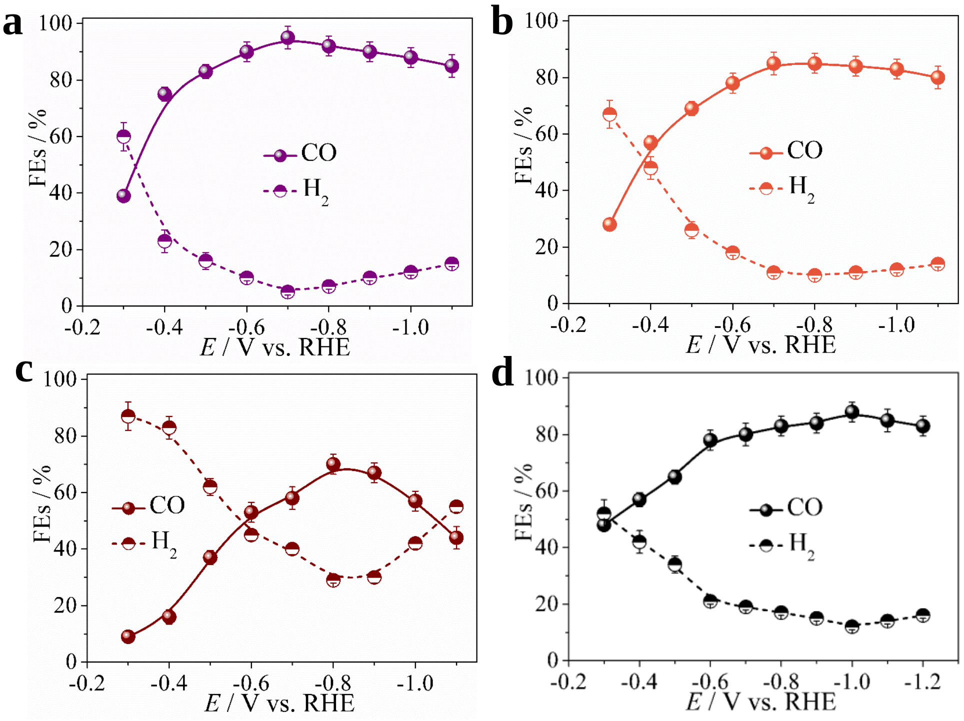

Supplementary Figure 15. **Summary of product Faradaic efficiencies.** Faradaic efficiencies of gas-phase products using P-NiSA/PCFM (a), P-NiSA/CFM (b), and P-PCFM (c) catalysts in H-type cell, respectively; Faradaic efficiencies using NiSA/PCFM in flow cell (d).

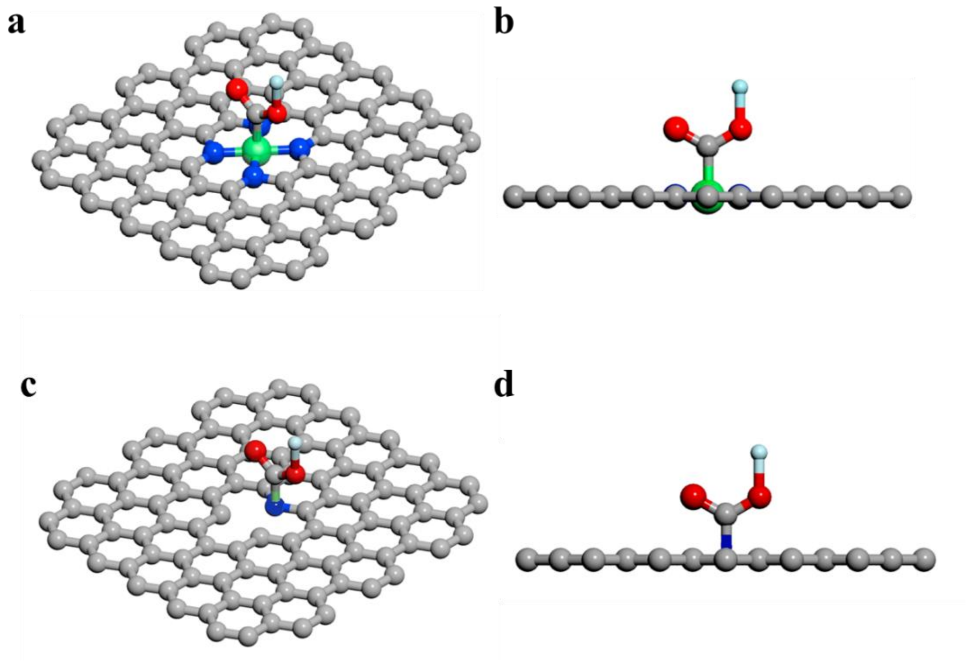

Supplementary Figure 16. **Structure model for calculation.** Structure model of Ni-N_4-C graphene (a–b) and N-C graphene (c–d). Green, gray, dark blue, red, and light blue spheres represent Ni, C, N, O, and H atoms, respectively.

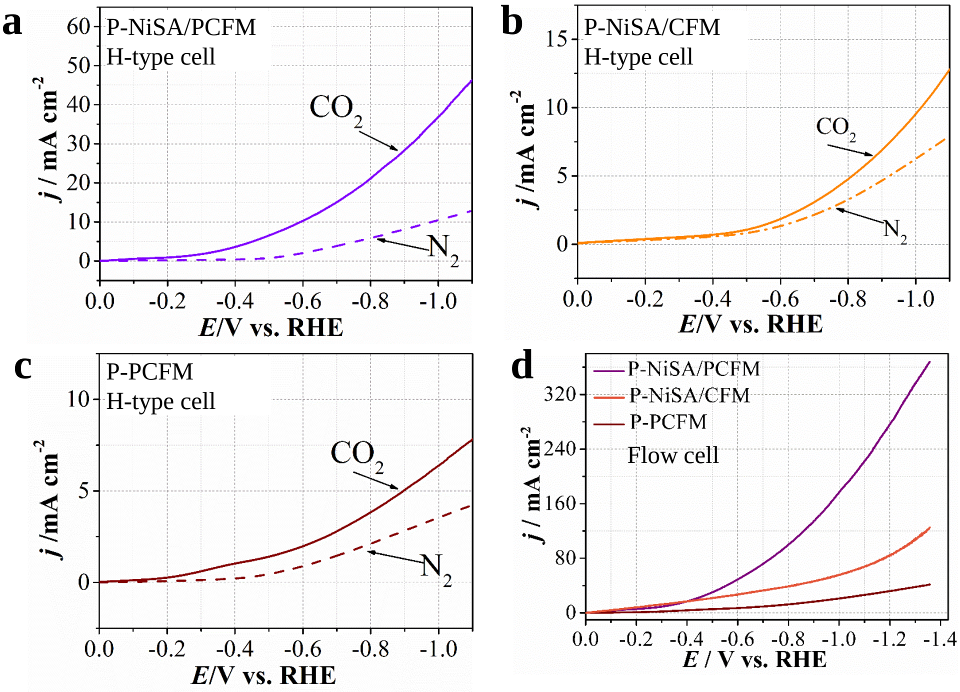

Supplementary Figure 17. **Summary of LSV tests.** LSV curves in CO_2-saturated (solid line) and N_2-saturated (dashed line) 0.5 M $KHCO_3$ solution in H-type cell using P-NiSA/PCFM (a), P-NiSA/CFM (b) and P-PCFM (c) catalysts, respectively; LSV tests of three samples in CO_2-saturated 0.5 M $KHCO_3$ solution in flow cell (d).

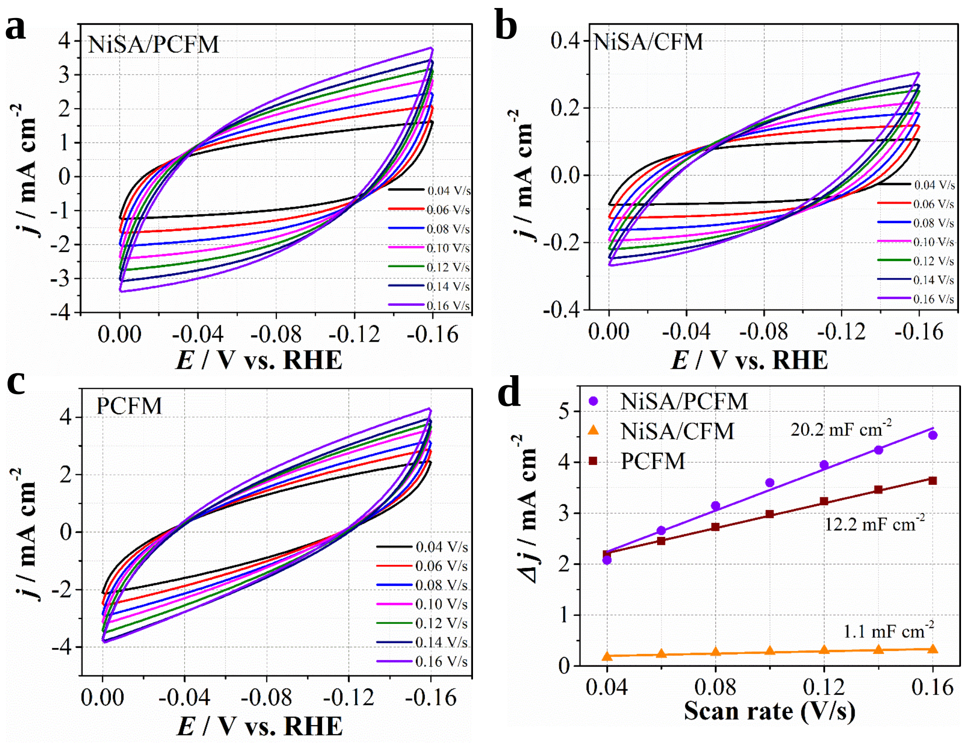

Supplementary Figure 18. **Summary of ECSA tests.** CVs of NiSA/PCFM (a), NiSA/CFM (b) and PCFM (c) from 0 to −0.16 V_{RHE} at various scan rates (0.04 to 0.16 V s^{-1}).

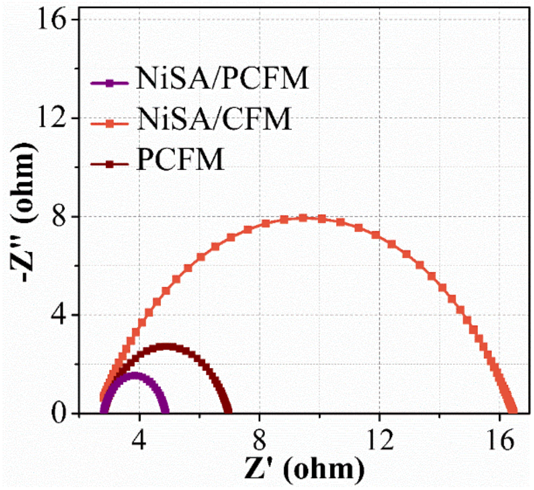

Supplementary Figure 19. **EIS tests.** EIS tests of three samples.

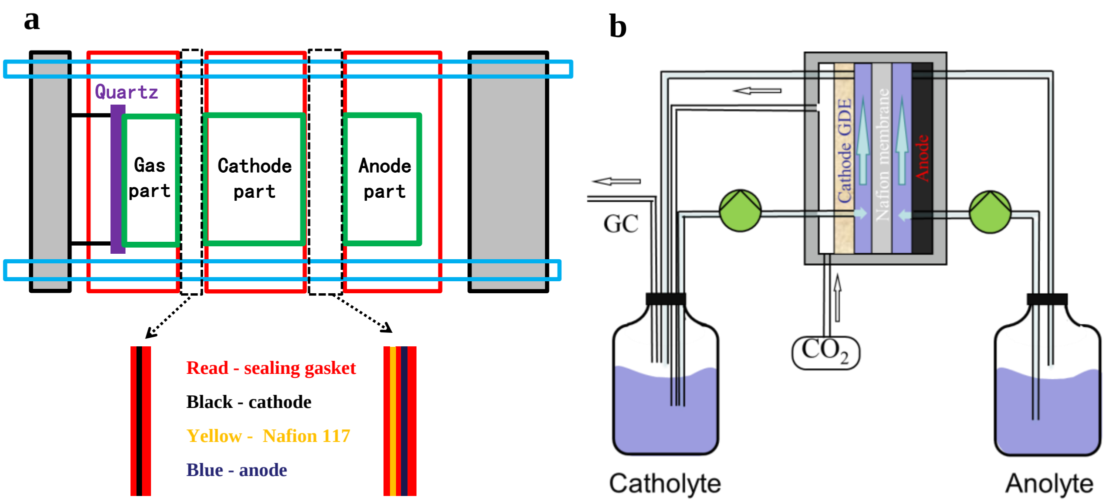

Supplementary Figure 20. **Schematic diagram of electrolysis device.** A flow cell device (a) and the whole CO_2 electrolysis system (b).

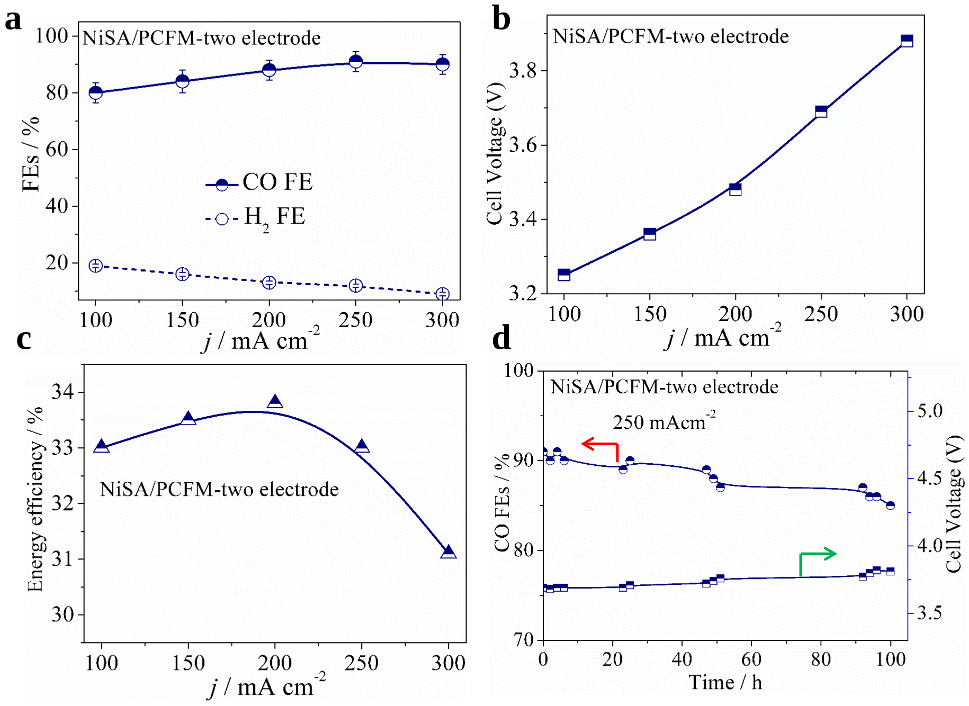

Supplementary Figure 21. CO_2 electrolysis of NiSA/PCFM membrane in two-electrode system. (a) CO faradic efficiency, (b) full-cell voltage, and (c) energy efficiency with different current densities; (d) Stability test at 250 mA cm^{-2} current density.

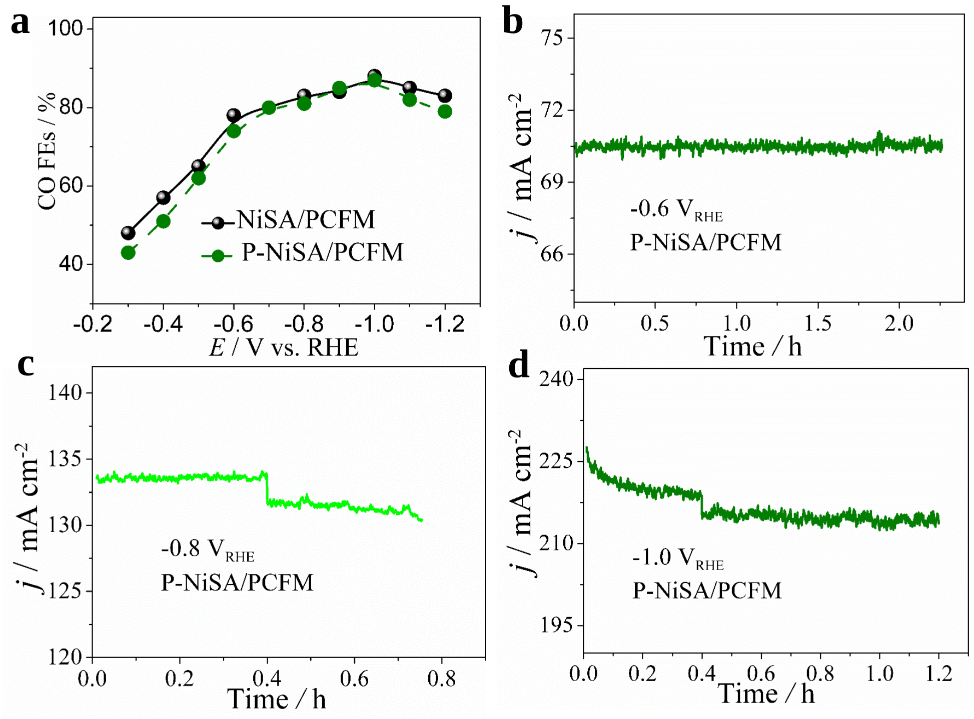

Supplementary Figure 22. CO_2 electrolysis using P-NiSA/PCFM. Faradaic efficiencies of CO using NiSA/PCFM and P-NiSA/PCFM (a); stability tests of P-NiSA/PCFM in flow cell at –0.6 V_{RHE} (b), –0.8 V_{RHE} (c) and –1.0 V_{RHE} (d).

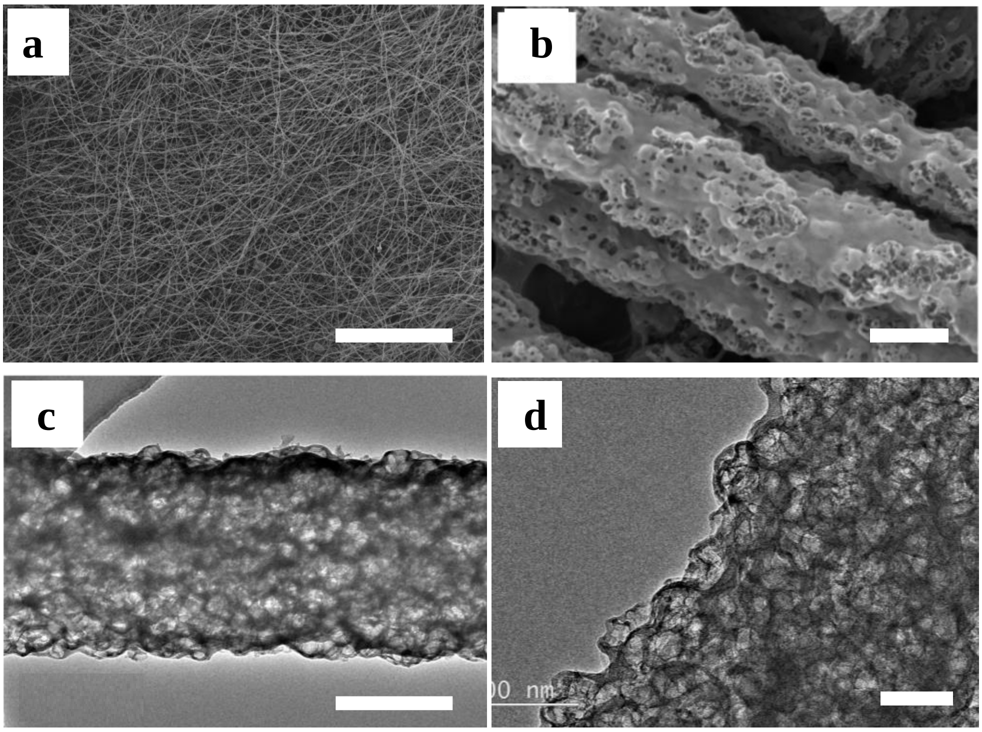

Supplementary Figure 23. Structural characterizations of NiSA/PCFM after electrolysis. (a−b) SEM and (c−d) TEM images of NiSA/PCFM.

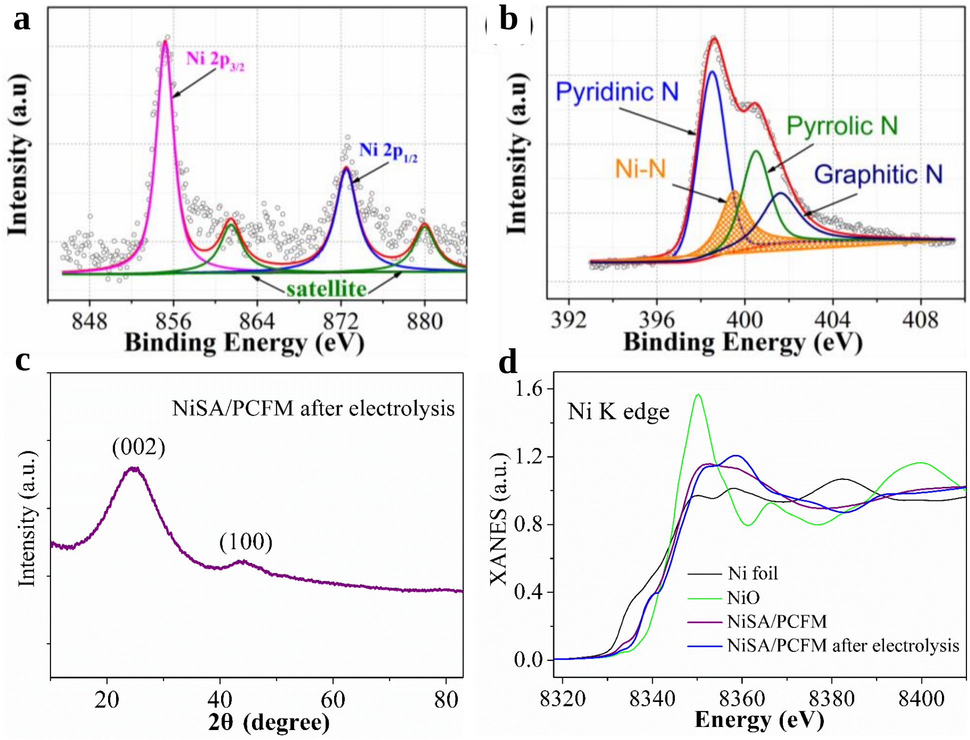

Supplementary Figure 24. Chemical characterizations of NiSA/PCFM after electrolysis. (a–b) XPS, (c) XRD and (d) XANES spectra of NiSA/PCFM.

Supplementary Table 1. Brunauer-Emmett-Teller data of various catalysts.

Entry	Specific surface area / $m^2\ g^{-1}$	Total pore volume / $cm^3\ g^{-1}$
NiSA/PCFM	714	1.6
NiSA/CFM	789	1.8
PCFM	723	1.6

Supplementary Table 2. The element percentage of three samples.

	**C（%）**	**N（%）**	**Zn（%）**	**Ni（%）**
NiSA/PCFM	87.53	7.86	-	0.85
NiSA/CFM	89.03	7.82	-	0.92
PCFM	90.28	8.25	-	-

Supplementary Table 3. Extended X-ray absorption fine structure fitting parameters at the nickel K-edge for various samples.

Sample	Shell	N [a]	R (Å) [b]	σ^2 (Å^2·10^3) [c]	ΔE_0 (eV) [d]	R factor (%)
NiSA/PCFM	Ni-N	4.1	1.93	8.5	–1.8	0.9
NiSA/CFM	Ni-N	3.9	1.93	8.5	–1.8	0.9

[a] N: coordination numbers; [b] R: bond distance; [c] σ^2: Debye-Waller factors; [d] ΔE_0: the inner potential correction. R factor: goodness of fit. S_0^2, 0.85, was obtained from the experimental EXAFS fit of NiPc reference by fixing CN as the known crystallographic value and was fixed to all the samples.

Supplementary Table 4. Calculated total energy, Zero Point Energy, and Gibbs free energy of each species.[a]

Type	E_{total} (eV)	ZPE (eV)	TS (eV)	G (eV)
CO_2	–22.95	0.31	0.66	–23.30
CO	–14.78	0.14	0.61	–14.32
H_2O	–14.23	0.58	0.67	–15.25
H_2	–6.77	0.28	0.41	–6.90

[a] ZPE: Zero Point Energy; T: temperature; S: entropy.

Supplementary Table 5. Electrocatalytic performance of recently reported single-atom catalysts for carbon dioxide reduction.

Cathode Materials	Atom%	Potential (vs.RHE)/V	Current Density/ mA cm^{-2}	Faradaic Efficiency / %	Products	Working Media	Loading/ mg cm^{-2}
**This work NiSA/PCFM**	**1.3**	**–1.0 (GDE) or –0.7 (H-type)**	**308.4 (GDE) or 56.1 (H-type)**	**88 (GDE) or 96 (H-type)**	**CO**	**0.5 M $KHCO_3$**	**1.0**
Ni SAs/N-C[1]	1.53	–0.89	10.48	71.9	CO	0.5 M $KHCO_3$	0.2
Ni-N_4-C[2]	1.41	–0.81	28.6	99.0	CO	0.5 M $KHCO_3$	0.02
Ni-NG[3]	0.44	–0.62	11	95.0	CO	0.5 M $KHCO_3$	1.0
NiN-GS[4]	-	–0.82	20	93.2	CO	0.5 M $KHCO_3$	1.0
A-Ni-NSG[5]	0.95	–0.61	22	98.0	CO	0.5 M $KHCO_3$	0.1
NiSA-N-CNTs[6]	20	–0.70	23.5	91.3	CO	0.5 M $KHCO_3$	0.2
Ni-SnS_2[7]	5	–0.90	19.6	93.0	formate/ CO	0.1 M $KHCO_3$	0.5
SE-Ni SAs@PNC[8]	-	–1.00	18.3	88	CO	0.5 M $KHCO_3$	0.4
Ni SAs/NCNTs[9]	6.63	–0.75	20	95	CO	0.5 M $KHCO_3$	0.8
Co-N_2[10]	-	–0.52	18.1	94.0	CO	0.5 M $KHCO_3$	0.8
Co-N_5[11]	3.54	–0.79	10.2	99.0	CO	0.2 M $NaHCO_3$	-
M-N_4[12]	-	–0.29/–0.38	-	93.0/45.0	CO	0.1 M $KHCO_3$	0.6
C- AFC©ZIF-8[13]	0.72	–0.63	10	93.0	CO	1 M $KHCO_3$	2.0
ZnN_x/C[14]	0.1	–0.43	4.8	95.0	CO	0.5 M $KHCO_3$	0.4
Sn-CF1000A D-Sn/N-C1000[15]	1.0	–0.8	11/5.12	62/91	formate/ CO	0.5 M $KHCO_3$ /0.1 M $KHCO_3$	0.97
Ni_3N/C[16]	-	–0.85	12	92.5	CO	0.1 M $KHCO_3$	0.75

Ni/N-CHSs[17]	-	–0.9	15	93.1	CO	0.5 M $KHCO_3$	0.5
Ni-NC-ATPA@C[18]	-	–0.7	6	93	CO	0.5 M $KHCO_3$	-
Fe^{3+}-N-C/GDE[19]	-	–0.45	94	＞90	CO	0.5 M $KHCO_3$	2.5
CoPc[20]	-	-	150	＞95	CO	1 M KOH	-
CoPc[21]	-	-	200	＞90	CO	water	0.2
Ni-NCB[22]	0.27	-	100	100	CO	0.5 M $KHCO_3$	1.25
H-CPs[23]	-	–1.0	48.66	97	CO	0.5 M $KHCO_3$	-

Supplementary Notes

In Supplementary Figure 6, N_2 sorption isotherms provided some information about the structures of two samples. NiSA/CFM displays a type I isotherm and pore size distribution for typical microporous structure (a–b). The hysteresis loop of type IV isotherm shows that the PCFM sample exhibits a mesoporous structure. The pore size distribution verifies that PCFM owns abundant micropores, mesopores and macropores (c–d).

In Supplementary Figure 21, we also conducted CO_2 electrolysis and stability test of NiSA/PCFM membrane in a flow cell device using two-electrode system without IR compensation. The range of constant current density was from 100 to 300 mA cm^{-2}. As revealed in Supplementary Figure 21a, the CO faradic efficiencies reached a maximum value of 91% at 250 mA cm^{-2} current density. 90% CO faradic efficiency could still be obtained at 300 mA cm^{-2} current density. The full-cell voltage increased from 3.25 V to 3.88 V along with the improvement of current density (Supplementary Figure 21b).

In addition, the Energy efficiency was calculated with the formula: Energy efficiency = $(E^o \times FE_{co})/E_{cell}$. FE_{co} is the Faradaic efficiency of CO, E_{cell} is the full-cell voltage. E^o was calculated with the formula: $E^o = E_{O2} - E_{CO} = 1.23\ V - (-0.11\ V) = 1.34\ V$, where E_{CO} is the CO_2/CO equilibrium potential, E_{O2} is the equilibrium potential of oxygen evolution reaction. The calculated energy efficiencies were displayed in Supplementary Figure 21c, and a maximal 33.8% energy efficiency was observed at 200 mA cm^{-2} current density.

Long-term electrolysis was performed using NiSA/PCFM membrane at a constant 250 mA cm^{-2} current density to investigate the stability. The CO faradic efficiency and cell voltage were detected during the test. As displayed in Supplementary Figure 21d, both CO faradic efficiency and cell voltage of NiSA/PCFM membrane only

declined slightly after 100 hours test, maintained more than 90% of initial value.

As revealed by the SEM and TEM images in Supplementary Figure 23, NiSA/PCFM retained network-like structure and well-distributed hollow nanocages within the carbonaceous nanofibers.

According to XPS spectra of NiSA/PCFM (Supplementary Figure 24a−b), the Ni $2p_{3/2}$ peak still locates between metallic Ni^0 (853.5 eV) and Ni^{2+} (855.8 eV), suggesting that the Ni atoms in NiSA/PCFM is likely to be in a low-valent state. XRD pattern of NiSA/PCFM (Supplementary Figure 24c) shows only two peaks centered at 26.2° and 44.0°, indexed to the (002) and (100) planes of carbon. No diffraction peaks of metallic Ni or Ni oxides are observed. Moreover, Supplementary Figure 24d exhibits the XANES spectrum in the Ni K-edge of NiSA/PCFM, using Ni foil and NiO as the references. The near-edge spectra of NiSA/PCFM after electrolysis locates between Ni foil and NiO, implying that the valence state of those isolated Ni atoms are between metallic (Ni^0) and oxidized (Ni^{2+}) status. Therefore, we can conclude that the Ni single atoms did not accumulate into nanoparticles during electrolysis.

Supplementary Methods

Characterizations. Linear sweep voltammograms (LSV) were performed at electrochemical workstation (Princeton Applied Research 263A). Potentiostatic electrolysis was recorded using a CHI 660C electrochemical Station (Shanghai Chenhua Instruments Company).

Liquid phase products were resolved by ^{1}H-NMR spectra recorded on an Ascend 400 (500 MHz, Bruker, Germany) spectrometer. Gas phase products were resolved by Gas Chromatography (SRI 8610C).

The morphologies were observed by a field emission scanning electron microscope (FE-SEM, FEI JEOL-7800F). Transmission electron microscopy (TEM) and highresolution TEM (HRTEM) images and element mapping analysis were obtained using JEM-2100F field emission electron microscope. High-angle annular dark-field scanning transmission electron microscopy (HAADF-STEM) measurements were performed on a JEOL JEM-ARF200F TEM/STEM with a spherical aberration corrector.

X-ray diffraction (XRD) was performed using a Rigaku MiniFlex 600 powder diffractometer with Cu Kα radiation (λ = 1.5406 Å).

N_2 adsorption/desorption was investigated by a Micromeritics ASAP 2460 instrument at 77 K, and the specific surface area were obtained by the Brunauer-Emmett-Teller (BET) equation. CO_2 adsorption was performed using Quantachrome Autosorb-IQ2-MP.

X-ray photoelectron spectra (XPS) were carried out on a ThermoVG Scientific ESCALAB 250 X-ray photoelectron spectrometer (Thermo Electron, U.K.) with Al Kα X-ray as the source.

XAFS measurements. The X-ray absorption find structure spectra (Ni K-edge) were collected at the beamline 1W1B station in Beijing Synchrotron Radiation Facility (BSRF). The storage rings of BSRF were utilized at 2.5 GeV with a maximum current

of 250 mA. Using Si(111) double-crystal monochromator, the data collection were performed in transmission mode with ionization chamber. All spectra were recorded at ambient conditions.

XAFS Analysis and Results. The acquired EXAFS data were processed based on the standard procedures using ATHENA module implemented in IFEFFIT software packages. The EXAFS data were achieved by subtracting post-edge background from overall absorption and normalizing to the edge jump step. Then, χ(k) data in k-space ranging from 2.6 to 12.6 $Å^{-1}$ were Fourier transformed to real (R) space with Hanning windows (dk = 1.0 $Å^{-1}$) to separate EXAFS contributions from different coordination shells. The quantitative information could be obtained by least-squares curve fitting in R space with the Fourier transform k space range of 2.6 to 12.6 $Å^{-1}$, with the module ARTEMIS of programs in IFEFFIT. The backscattering amplitude F(k) and phase shift Φ(k) were calculated using the FEFF8.0 code.

Synthesis of ZIF-8. ZIF-8 crystals were prepared via rapidly pouring an aqueous solution (16 mL deionized water) of $Zn(NO_3)_2·6H_2O$ (7.9 mmol, 2.34 g) into an aqueous solution (160 mL deionized water) of 2-methylimidazole (553 mmol, 45.4 g). Then, the mixed solution magnetically stirred for five minutes at room temperature. After stirring, the mixture was filtered by filter paper. The liquid product was collected by centrifuge (7000 revolutions per minute, 30 minutes) and thoroughly cleaned by deionized water for at least five times. The product was dried at 60 ^{o}C for 12 hours in a drying oven.

Synthesis of NiSA/PCFM, NiSA/CFM and PCFM. NiSA/PCFM were synthesized using an electrospinning process. First, 1.5 g of polyacrylonitrile (PAN, Mw =130000), 1.5 g of ZIF-8 and 30 mg of $Ni(NO_3)_2·6H_2O$ powder were dissolved in 15 mL of N, N-dimethylformamide (DMF) via vigorously stirring to get a homogenous mixture. The transparent mixture was diverted into a plastic syringe with a stainless needle at the tip. The needle was connected to a high voltage power supply. The voltage power, solution flow rate and spin distance were 20 kV, 0.7 mL h^{-1} and 12 cm, respectively. All experiments were carried out at room temperature.

After electrospinning procedure, the as-spun raw fibers were firstly preoxidated in air at 250 °C for 1 hour and immediately carbonized under argon gas at an optimum temperature of 900 °C for 2 hours. The heating rate was 5 °C min^{-1}. After heat treatment, the residue was cooled to room temperature under argon gas flow. Then, the resultant materials were immersed into H_2SO_4 solution (3.0 M) for 10 hours to remove the remaining Zn species. NiSA/PCFM membrane was thus obtained.

The precursor solution containing 1.5 g of PAN, 30 mg $Ni(NO_3)_2{\cdot}6H_2O$ and no ZIF-8 was also electrospun and carbonized under 900 °C to act as control for the comparison. The resulting product was referred to as NiSA/CFM.

The precursor solution containing 1.5 g of PAN and 1.5 g of ZIF-8 nanoparticles was also electrospun and carbonized under 900 °C, the resulting product was referred to as PCFM.

CO_2 Reduction Procedure in H-type cell. Electrolysis and LSVs was performed in a in conventional H-type electrochemical cell separated by Nafion®117 membrane between cathode and anode, consisted of Pt foil as the counter electrode and an Ag/AgCl as reference electrode in CO_2 or N_2-saturated 0.5 M $KHCO_3$ solution. A specific volume of the catalyst (PCFM, NiSA/CFM or NiSA/PCFM) ink was then drop-casted on carbon paper electrode to achieve an approximately 1 mg cm^{-2} loading amount and then dried at room temperature. This carbon paper would be utilized as the cathode for CO_2 electro-reduction. The catalyst ink was prepared via the following steps: specific amount catalyst powder (like NiSA/PCFM) was added into a mixture solution of 110 µL of Nafion solution (5 wt%, Dupond) and 890 µL of ethanol. The mixed solution was sonicated for 30 min to form a homogeneous catalyst ink.

CO_2 Reduction Procedure in GDE device. Measurements at high current densities were performed in a self-made micro flow cell. Different from tests using H-type cell, NiSA/PCFM membranes were cut into the desired size (4 cm^2) and shape and directly served as cathode compartment, since these membranes are flexible and self-supporting. Certain amount of Nafion solution (5 wt%, Dupond) was spray-coated on this membrane to get a hydrophobic property. A commercial Pt/C electrode was used as anode and an Ag/AgCl were acted as the reference. 0.5 M

$KHCO_3$ aqueous solution was utilized as electrolyte, which were separated by a piece of Nafion®117 membrane. Electrolyte were cycled at with a rate of 50 mL min^{-1}. The CO_2 gas was supplied to the cathode and was flown through the NiSA/PCFM membrane at rate of 20 mL min^{-1}. A specific volume of NiSA/PCFM ink was also drop-casted on a gas diffusion layer (SIGRACET) as the cathode compartment in flow cell for comparison, which was denoted as P-NiSA/PCFM.

All potentials in this paper were converted to reversible hydrogen electrode (RHE) by the following Nernst equation:

$$E(RHE) = E(Ag/AgCl) + 0.199 + 0.059 \times pH \qquad (1)$$

The electrolyzer outlet was vented directly into the gas-sampling loop of the gas chromatograph (SRI-8610C) equipped with a flame ionization detector (FID) and a thermal conductivity detector (TCD). The Faradaic efficiency of CO (or H_2) was calculated with the equation:

$$FE = n_{CO} \times F \times m_{CO} \times V_m / I \qquad (2)$$

Where n_{CO} is the number of electrons exchanged, F = 96,485 C mol^{-1}, m_{CO} is the mole fraction of CO in the gaseous mixture analysed, V_m is the molar flow rate in mol s^{-1}, and I is the total current in A. The molar flow rate is derived from the volume flow rate V by the relation $V_m = pV/RT$. p is the atmospheric pressure in Pa, R is 8.314 J mol^{-1} K^{-1}, and T is the temperature in K.

DFT Calculations. The calculations in this manuscript were performed using VASP package with PBE functionals. Each structure was fully relaxed until all forces were lower than 0.01 eV/Å. A 3×3×1 K-point mesh was used, and the vacuum layer was set to 14 Å. The lattice constants along with x, y and z directions are 12.68 Å, 12.34 Å and 15.00 Å, respectively.

The adsorption energy was calculated by the following equation:

$$E_a = E_{final} - E_{substrate} - E_{adsorbate} \qquad (3)$$

where E_a, E_{final}, $E_{substrate}$ and $E_{adsorbate}$ are the adsorption energy of adsorbate (*COOH)

on substrate (N- and Ni-doped graphene structure), total energy of adsorbate on this substrate, total energy of substrate, and total energy of adsorbate.

The free energy of this reaction was calculated by following equation:

$$\Delta G = E_a + \Delta ZPE - T\Delta S \qquad (4)$$

where ΔG, ΔZPE and ΔS represent Gibbs free energy, zero point energy and the entropy, respectively.

Supplementary Reference

1. Zhao, C. M. et al. Ionic exchange of metal-organic frameworks to access single nickel sites for efficient electroreduction of CO_2. *J. Am. Chem. Soc.* **139**, 8078–8081 (2017).

2. Li, X. G. et al. Exclusive Ni-N_4 sites realize near-unity CO selectivity for electrochemical CO_2 reduction. *J. Am. Chem. Soc.* **139**, 14889–14892 (2017).

3. Jiang, K. et al. Isolated Ni single atoms in graphene nanosheets for high-performance CO_2 reduction. *Energy. Environ. Sci.* **11**, 893–903 (2018).

4. Jiang, K. et al. Transition-metal single atoms in a graphene shell as active centers for highly efficient artificial photosynthesis. *Chem* **3**, 950–960 (2017).

5. Yang, H. B. et al. Atomically dispersed Ni(i) as the active site for electrochemical CO_2 reduction. *Nat. Energy* **3**, 140–147 (2018).

6. Cheng, Y. et al. Atomically dispersed transition metals on carbon nanotubes with ultrahigh loading for selective electrochemical carbon dioxide reduction. *Adv. Mater.* **30**, 1706287 (2018).

7. Zhang, A. et al. Nickel doping in atomically thin tin disulfide nanosheets enables highly efficient CO_2 reduction. *Angew. Chem. Int. Ed.* **57**, 10954–10958 (2018).

8. Yang, J. et al. In situ thermal atomization to convert supported nickel nanoparticles into surface-bound nickel single-atom catalysts. *Angew. Chem. Int. Ed.* **57**, 14095–14100 (2018).

9. Lu, P. et al. Facile synthesis of single-nickel-atomic dispersed N-doped carbon framework for efficient electrochemical CO_2 reduction, *Appl. Catal.-B: Environ.* **241**, 113–119 (2019).

10. Wang, X. et al. Regulation of coordination number over single Co sites: triggering the efficient electroreduction of CO_2. *Angew. Chem. Int. Ed.* **57**, 1944–1948 (2018).

11. Pan, Y. et al. Design of Single-atom Co-N_5 catalytic Site: a robust electrocatalyst for CO_2 reduction with nearly 100% CO selectivity and remarkable stability. *J. Am. Chem. Soc.* **140**, 4218–4221 (2018).

12. Pan, F. et al. Unveiling active sites of CO_2 reduction on nitrogen-coordinated and atomically dispersed iron and cobalt catalysts. *ACS Catal.* **8**, 3116–3122 (2018).

13. Ye, Y. et al. Surface functionalization of ZIF-8 with ammonium ferric citrate toward high exposure of Fe-N active sites for efficient oxygen and carbon dioxide electroreduction. *Nano Energy* **38**, 281–289 (2017).

14. Yang, F. et al. Highly efficient CO_2 electroreduction on ZnN_4-based single-atom catalyst. *Angew. Chem. Int. Ed.* **57**, 12303–12307 (2018).

15. Zhao, Y., Liang, J., Wang, C., Ma, J. & Wallace, G. G. Tunable and efficient tin modified nitrogen-doped carbon nanofibers for electrochemical reduction of aqueous carbon dioxide. *Adv. Energy Mater.* **8**, 1702524 (2018).

16. Moller, T. et al. Efficient CO_2 to CO electrolysis on solid Ni-N-C catalysts at industrial current densities. *Energy Environ. Sci.* **12**, 640–647 (2019).

17. Yuan, C. Z. et al. Tuning the activity of N-doped carbon for CO_2 reduction via in situ encapsulation of nickel nanoparticles into nano-hybrid carbon substrates. *J. Mater.*

Chem. A **7**, 6894-6900 (2019).

18. Jia, M. W. et al. Carbon-supported Ni nanoparticles for efficient CO_2 electroreduction. *Chem. Sci.* **9**, 8775–8780 (2018).

19. Gu, J., Hsu, C. S., Bai, L., Chen, H. M. & Hu, X. Atomically dispersed Fe^{3+} sites catalyze efficient CO_2 electroreduction to CO. *Science* **364**, 1091–1094 (2019).

20. Ren, X. et al. Molecular electrocatalysts can mediate fast, selective CO_2 reduction in a flow cell. *Science*, **365** 367–369 (2019).

21. Z. L. Yin, et al. An alkaline polymer electrolyte CO_2 electrolyzer operated with pure water. *Energy Environ. Sci.* **12**, 2455–2462 (2019).

22. Zheng, T. T. et al. Solid-diffusion synthesis of single-atom catalysts directly from bulk metal for efficient CO_2 reduction. *Joule* **3**, 584–594 (2019).

23. Zheng, T. et al. Large-scale and highly selective CO_2 electrocatalytic reduction on nickel single-atom catalyst. *Joule* **3**, 265–278 (2019).
